# Top-down feedback normalizes distortion in early visual word recognition: Insights from masked priming

**DOI:** 10.3758/s13423-024-02585-2

**Published:** 2024-10-18

**Authors:** Maria Fernández-López, Olga Solaja, Davide Crepaldi, Manuel Perea

**Affiliations:** 1 Department of Basic Psychology, Universitat de València, Av. Blasco Ibáñez, 21, 46010 València, Spain; 2 Scuola Internazionale Superiore di Studi Avanzati (SISSA), Trieste, Italy; 3 Department of Methodology and ERI-Lectura, Universitat de València, València, Spain; 4 CINC, Universidad Nebrija, Madrid, Spain

**Keywords:** Lexical access, Models of word recognition, Masked priming

## Abstract

The identification of visually presented words tolerates distortions in the input format, as Hannagan et al. *Plos One, 7,* e32121, (2012) demonstrated in a masked priming lexical decision task, showing sizable identity-priming effects with CAPTCHA-like primes. This tolerance to distortion has two potential explanations: bottom-up normalization in the encoding stage (Dehaene et al., *Trends in Cognitive Sciences, 9,* 335–341, 2005) or top-down lexical feedback (McClelland & Rumelhart, *Psychological Review*, *88*, 375–407, 1981). To disentangle the predictions of these accounts, we conducted two masked identity-priming experiments with printed and CAPTCHA-like distorted primes on high- and low-frequency words. The rationale was that, in the distorted format, high-frequency words would benefit more from top-down feedback than low-frequency words. Results in the lexical decision experiment showed that, for high-frequency words, identity-priming effects were only slightly greater for printed than for CAPTCHA-like primes, whereas this difference was larger for low-frequency words. In contrast, when employing the same-different matching task, which does not require lexical access, the identity-priming effect was greater for printed primes and was unaffected by word frequency. Thus, during lexical access, top-down feedback may help normalize the visual input in the early stages of word recognition, challenging bottom-up models of visual word recognition.

**Supplementary Information:**

The online version contains supplementary material available at 10.3758/s13423-024-02585-2.

## Introduction

An aspect often overlooked in casual observation when we read a text, be it a journal article, an email, a newspaper, an advertisement, or a street sign, is that we are exposed to large perceptual variations in the printed words (e.g., compare gas and gas; see Wong et al., 2018). Interestingly, aside from special cases (e.g., *script* or decorate fonts), the reading process remains largely invariant across fonts (Rayner et al., 2006). Neurobiologically inspired models of written word recognition explain this invariance with the assumption that, during lexical access, the visual input is progressively filtered by a hierarchical series of neuron layers (e.g., Local Combination Detector (LCD) model; Dehaene et al., 2005; see also Grainger et al., 2008). The neurons that compose these layers, tolerant to minor perceptual variations, map the visual input onto abstract representations of the word’s constituent letters stored in lexical memory.

An excellent demonstration of the abilities of the human word-recognition system to rapidly discard variations in the visual input was provided by Hannagan et al. (2012). They conducted a masked priming lexical decision experiment, in which after a forward mask, a briefly presented, 50-ms prime in printed or distorted format (e.g., chair,  ) preceded the target stimuli. Primes could be identical or unrelated to the printed target words, thus allowing them to examine the differences in identity-priming effect across formats (e.g., printed primes: chair*-*CHAIR vs. olive*-*CHAIR; distorted primes:  -CHAIR vs.  -CHAIR). While the identity-priming effect was larger when the primes were printed rather than in a distorted format, the effect was sizable for distorted primes. Hannagan et al. (2012) concluded that the human ability to solve distorted stimuli builds on a high degree of tolerance to changes in the letter forms. Critically, given that the primes were presented very briefly, these findings reflect that this tolerance does not occur at late processing stages where readers could consciously decipher these distorted stimuli but rather during the initial stages of visual word recognition (see Fernández-López et al., 2023, and Gil-López et al., 2011, for similar evidence with rotated and handwritten primes).

Two potential explanations can capture the tolerance to letter distortion in the first moments of word processing. On the one hand, one might argue that the normalization of the noisy visual input occurs mainly bottom-up during the letter encoding stage. As proposed by the LCD model of visual word recognition (Dehaene & Cohen, 2007; Dehaene et al., 2005), letter detectors in the word recognition system can be enabled by minor variations in the shape of the letter forms in a feedforward direction. On the other hand, in the framework of fully interactive models of visual word recognition (McClelland & Rumelhart, 1981; see also Carreiras et al., 2014), at least part of this resilience to variability occurs via top-down lexical feedback that helps regularize the perceptual representation of the distorted stimuli.

There is some empirical evidence with the masked priming lexical decision task that has been taken to support interactive over feedforward models of visual word recognition. Lexical decision times for identity prime-target nonword pairs are faster when they keep the same letter case (e.g., DIUSE*-*DIUSE) than when they are in different letter case (e.g., diuse*-*DIUSE). However, this difference is absent for words (house*-*HOUSE produces similar response times as HOUSE*-*HOUSE; see Jacobs et al., 1995, and Perea et al., 2015, for behavioral evidence; see Gutierrez-Sigut et al., 2019, and Vergara-Martínez et al., 2015, for electrophysiological evidence). If the access to the abstract representations only occurs bottom-up, one would expect a similar pattern of priming effects for both words and nonwords. However, there is an interpretive issue with this rationale: words and nonwords in the lexical decision task differ not only on whether only words have a lexical representation but also in the responses they elicit (“yes” vs. “no”) – note that “no” responses in lexical decision tasks can be made using different sources of information than “yes” responses (Davis, 2010; Dufau et al., 2012).

A more definitive conclusion supporting the idea that top-down feedback affects the initial stages of lexical processing could be achieved by comparing two categories of word stimuli in a masked priming lexical decision task. In the present experiments, we included word frequency as a factor (half of the words were high-frequency, and the other half were low-frequency). The premise was that top-down lexical feedback would benefit more the identity of CAPTCHA-like primes when derived from high-frequency than low-frequency words – note that the lexical units from high-frequency words are activated more rapidly than those of low-frequency words (see Davis, 2010), and this activation may, at least partly, compensate for the effect of letter distortion (see Grainger et al., 2012). Therefore, this manipulation allowed us to directly test whether the normalization of letter distortion is shaped by lexical feedback.

**Fig. 1:**
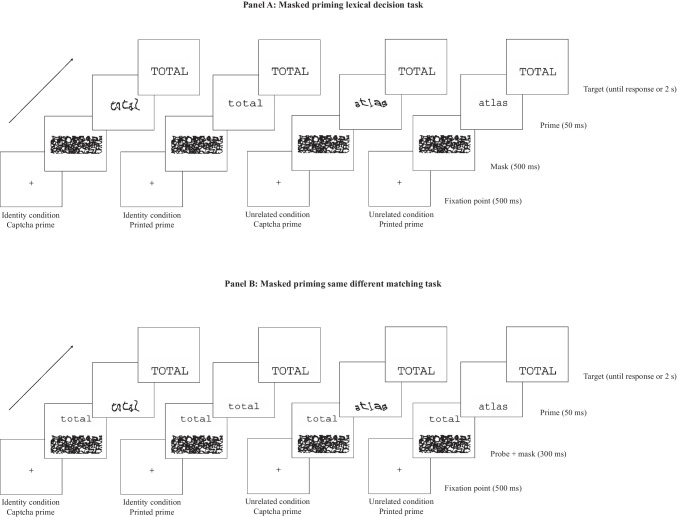
Illustration of the sequence of events in the masked priming lexical decision task (word targets; panel **A**) and the masked priming same-different matching task (same trials; panel **B**)

Thus, the main goal of the experiments was to resolve the theoretical question of the role of top-down processing during the early phases of word processing. To that end, we designed two masked priming experiments comparing masked identity-priming effects with printed versus CAPTCHA-like primes for high- and low-frequency words. Experiment 1 used a masked priming lexical decision task (see Fig. 1A). In this task, participants must access the lexicon to perform adequately, as nonword foils are orthographically legal and matched on sublexical characteristics with the word targets. As stated earlier, top-down lexical feedback appears to affect the masked priming lexical decision task – at least when comparing word versus nonword targets (Jacobs et al., 1995; Vergara-Martínez et al., 2015). As a control, Experiment 2 employed the masked priming paradigm with a task designed to only engage prelexical processes: the same-different matching task (Norris & Kinoshita, 2008; Perea et al., 2016; see Fig. 1B). This task compares a probe and a target, allowing participants to rely on visual or orthographic information without accessing the lexicon to perform it properly.

We can envision two potential scenarios for Experiment 1 (lexical decision task). The first scenario would correspond to feedforward models of visual word recognition. These models rely on bottom-up activation from the visual input, in which the letter detectors have some tolerance to changes in letter form (LCD model; Dehaene et al., 2005). In this case, one would only expect a smaller identity-priming effect for CAPTCHA-like than for printed primes (Hannagan et al., 2012), regardless of word frequency. The second scenario would correspond to fully interactive models of visual word recognition, in which top-down processes from the lexical level would modulate lower levels of processing even in the initial stages of visual word identification. In this latter scenario, the higher level of activation elicited by high-frequency words in the initial moments of word processing (Grainger et al., 2012) may partially outweigh the detrimental effects of letter distortion. Therefore, the reading cost caused by the visual format of the identity primes (CAPTCHA-like vs. printed) would play a less prominent role for high- than low-frequency words, thus predicting a three-way interaction (prime-target relation × word frequency × prime format).^1^ We defer the justification of Experiment 2 until later.

## Experiment 1: Lexical decision task

### Method

The study (hypothesis, analyses, exclusion criteria, and sample size justification) was preregistered at OSF

#### Participants

We recruited 240 native speakers of Spanish (mean age = 28 years old, range: 19–40 years), with normal (corrected) vision and no language-related or literacy-related disorders, via Prolific (https://www.prolific.com). This sample size allowed us to obtain 3,600 observations per condition, above Brysbaert and Stevens’s (2018) suggestion to capture small effects in masked priming experiments. All participants signed a consent form before the experiment and were paid according to the standards of the Prolific platform. The experiments were approved by the Ethics Committee for Experimental Research of the University of València, following the guidelines of the Helsinki Declaration.

#### Materials

For both experiments, we selected 240 word targets (120 high-frequency and 120 low-frequency targets) from the subtitle corpus of the EsPal Spanish database (Duchon et al., 2013). For the high-frequency targets, the average Zipf frequency was 5.14 (range 4.69–6.11), and the average OLD20 was 1.43 (range 1–1.95); for the low-frequency targets, the average Zipf frequency was 3.26 (range 2.17–3.70), and the average OLD20 was 1.47 (range 1–1.95).^2^Of this stimulus set, 120 targets (60 high-frequency and 60 low-frequency) were used in Experiment 1. In contrast, the remaining 120 were used in Experiment 2 (i.e., each participant was exposed to all 240 word targets but in different tasks – this was counterbalanced). In Experiment 1 (lexical decision task), each target word (e.g., MUNDO [world]) was paired with an identity prime (e.g., mundo) or an unrelated prime (e.g., silla [chair]). To act as unrelated primes, we selected 120 words with an average Zipf frequency of 4.20 (range 3.70–4.72) and an average OLD20 of 1.40 (range 1–1.85) (Duchon et al., 2013). To act as foils in the lexical decision task, we created 240 orthographically legal nonwords matched with the words in sublexical characteristics with Wuggy (Keuleers & Brysbaert, 2010). Each target nonword (e.g., BOMPA) was paired with an identity prime (e.g., bompa) or an unrelated prime (e.g., urián). Distorted items were generated using a script written in Python (Python version 3.6.6; packages: pandas (version 1.1.5), PIL (version 8.0.1)), which generated a wave-like distortion for each letter string (the script is available via the Open Science Framework (OSF) repository). We created eight lists to counterbalance the prime–target combinations across different conditions and the two tasks (lexical decision and same-different matching tasks).

#### Procedure

The script was written in PsychoPy3 Builder v2020.2.10 (Peirce & Macaskill, 2022) and was conducted online using the Pavlovia platform (www.pavlovia.org) (see Angele et al., 2023, for a demonstration of the validity of the use of online masked priming with PsychoPy). Each trial of the lexical decision task began with a mask made of several overlayed CAPTCHA-like items from the practice trials (as the Hannagan et al. (2012) experiment) displayed for 500 ms in the center of a computer screen. The mask was followed by a 50-ms printed/CAPTCHA-like prime stimulus, which, in turn, was replaced by the uppercase target stimulus presented until a response was made or 2 s had elapsed (in case of not responding before the timeout, the responses were categorized as an error) (see Fig. 1a). Participants were instructed to decide whether the uppercase stimulus was a word or not by pressing the “M” key (word) or the “Z” key (nonword) with their index fingers. The instructions stressed both speed and accuracy and did not mention the existence of any briefly presented primes. A total of 16 practice trials preceded the 240 experimental trials. Participants were randomly assigned to one of the eight counterbalancing lists of the study. Moreover, the sequence of trials was presented randomly for each participant. This task took approximately 13–15 min.

#### Data analyses

The independent variables were (1) prime-target relation (identity vs. unrelated), (2) word frequency (high vs. low), and (3) prime format (printed vs. CAPTCHA-like). The dependent variables were (correct) response time (RT) and accuracy. The correct RT and accuracy data were modeled with Bayesian linear mixed-effects models in R (R Core Team, 2022) using the *brms* package (Bürkner, 2021). The fixed effects were prime-target relation (identity vs. unrelated), word frequency (high vs. low), and prime format (printed vs. CAPTCHA-like). In all cases, the encoding was -0.5 and 0.5 (i.e., effect coding). We employed the default non-informative parameters of the *brms* package – this allows the posterior distributions of the parameters to be essentially shaped by the observed data (Bürkner, 2021). We used the ex-Gaussian family function to model the RT data, capturing the Gaussian (*mu*) and exponential (*beta,* 1/λ;λ is the rate parameter of the exponential distribution and models the tail of long RTs) components of latency distribution across conditions, and the Bernoulli family function to model the accuracy data (correct responses correspond to 1 and error responses to 0). The models were the maximal in terms of random-effect structure:

$$\mathrm{RT}\;\left(\mathrm{or}\;\mathrm{accuracy}\right)\;=\;\mathrm{relation}\;\ast\;\mathrm{frequency}\;\ast\;\mathrm{format}\;+\;\left(1+\mathrm{relation}\;\ast\;\mathrm{frequency}\;\ast\;\mathrm{format}\;\left|\;\mathrm{subject}\right.\right)\;+\;\left(1+\;\mathrm{relation}\;\ast\;\mathrm{format}\;\left|\;\mathrm{item}\right.\right)$$

We ran 5,000 iterations across four chains – 1,000 for warm-up. Bayesian linear mixed-effects models indicate an estimate of the parameters (the median of their posterior distributions) and their 95% credible intervals (CrIs) based on the posterior distributions rather than *p*-values. We interpret evidence of a main effect or interaction if its 95% CrI does not overlap zero.

### Results and discussion

Error rates were 5.58% for word trials and 4.66% for nonword trials. We focused on the word targets because our manipulation relied on whether the identity-priming effects with CAPTCHA-like and printed primes interacted with a lexical factor (word frequency). RTs below 250 ms (0.27%) were excluded from correct RT analyses. Table 1 displays the mean RT and error rates in each condition, and Table 2 presents the estimates of the posterior distributions for the RTs. The accuracy analyses are available in the Online Supplemental Material (OSM; Table 5).

**Table 1:** Mean correct response times (RTs, in ms), percent error rates, and standard errors (SEs, in brackets) for words in Experiment 1 (masked priming lexical decision task)

		High-frequency		Low-frequency	
		RT (SE)	% Error (SE)	RT (SE)	% Error (SE)
Printed	Related	576 (7.67)	1.3 (0.705)	638 (9.16)	7.0 (1.54)
	Unrelated	608 (7.67)	2.5 (0.966)	684 (9.23)	11.1 (1.92)
Captcha	Related	584 (7.55)	1.8 (0.830)	662 (9.61)	2.1 (1.67)
	Unrelated	607 (7.60)	2.3 (0.929)	681 (8.89)	10.5 (1.90)
Printed	Priming effect	32	1.2	46	4.1
Captcha		23	0.5	19	2.4

*Note:* SEs were within-participant SEs around the mean (Cousineau & O’Brien, 2014)

**Table 2:** Posterior estimates parameters, estimation errors, and 95% credible intervals for the fixed effects of the model fitted for the response times to word targets in the lexical decision task (Experiment 1)

Parameters	Estimation	Estim. Error	Lower bound	Upper bound
Intercept (μ)	628.31	4.28	620.04	636.82
Intercept (β)	4.61	0.02	4.56	4.65
**Relatedness (**μ**)**	**31.61**	**1.65**	**28.37**	**34.82**
**Format (**μ**)**	**-6.03**	**1.38**	**-8.72**	**-3.33**
**Word-Frequency (**μ**)**	**71.02**	**3.55**	**64.13**	**78.01**
**Relatedness x Format (**μ**)**	**16.83**	**2.66**	**11.59**	**22.06**
**Relatedness x Word-Frequency (**μ**)**	**7.11**	**3.00**	**1.30**	**12.93**
Format × Word-Frequency (μ)	-1.96	2.70	-7.20	3.33
**Relatedness × Format × Word-Frequency (**μ**)**	**14.93**	**5.20**	**4.68**	**25.17**
**Relatedness (β)**	**0.03**	**0.02**	**0.00**	**0.06**
Format (β)	0.00	0.02	-0.03	0.03
**Word-Frequency (β)**	**0.34**	**0.02**	**0.29**	**0.39**
Relatedness × Format (β)	0.00	0.03	-0.06	0.07
Relatedness × Word-Frequency (β)	0.00	0.03	-0.06	0.07
Format × Word-Frequency (β)	0.01	0.03	-0.05	0.07
**Relatedness × Format × Word-Frequency (β)**	**0.16**	**0.07**	**0.03**	**0.29**

*Note:* The estimations in bold indicate that the 95% Credible Interval did not overlap with zero

#### Response times

As shown in Table 2, analysis of the Gaussian component showed faster RTs for identity than for unrelated pairs, for high-frequency than low-frequency words, and for targets with printed primes than for CAPTCHA-like primes. Critically, we found evidence that the identity-priming effect was jointly modulated by prime format and word frequency (three-way interaction; *b* = 14.93, 95% CrI [4.68, 25.17]): for high-frequency words, the identity-priming effect was only 9 ms greater for printed than for CAPTCHA-like primes (32 vs. 23 ms, respectively), whereas for low-frequency words this difference increased to 25 ms (46 vs. 19 ms, for printed and CAPTCHA-like primes, respectively). The distributional differences are visualized as delta plots in the OSM.

The analyses of the RT model on the exponential component showed evidence of effects of identity priming (*b* = 0.29, 95% CrI [0.00, 0.06]) and word frequency (*b* = 0.34, 95% CrI [0.29, 0.38]), together with a three-way interaction (*b* = 0.16, 95% CrI [0.03, 0.29]) that reflected the same direction as the Gaussian component (see Table 2).

The critical finding in the present experiment was the presence of an interaction between prime-target relation, prime format, and word frequency. This interaction revealed that the difference in magnitude of the identity-priming effect for printed and CAPTCHA-like primes was smaller for high-frequency words (32 vs. 22 ms for identity and primes, respectively) than for low-frequency words (46 vs. 19 ms for printed and CAPTCHA-like primes, respectively).

Overall, this outcome favors interactive models of visual word recognition that assume that lexical top-down feedback may normalize early letter-encoding processes in distorted stimuli, benefitting more high-frequency words.

## Experiment 2: Same-different matching task

We conducted a second experiment, using a masked priming same-different matching task with the same group of participants and basic experimental design as Experiment 1. This task was designed to tap prelexical processing (Norris & Kinoshita, 2008). Unlike in the lexical decision task, RTs appear to be only minimally faster for high- than for low-frequency words (8.5 ms, *p* = .09), suggesting that lexical involvement is minimal (Norris & Kinoshita, 2008; see Perea et al., 2016, for converging evidence; but see Kelly et al., 2013, for higher-level effects with auditory presentations^3^).

The logic of Experiment 2 is straightforward. If the masked priming same-different matching task only taps prelexical processing, we expect the effects of identity priming to be additive with word frequency (i.e., negligible top-down lexical feedback) – indeed, we would expect a minimal effect of word-frequency in light of Norris and Kinoshita’s (2008) null finding. Conversely, any evidence of a modulating effect of word frequency on the size of identity-priming effects would challenge Norris and Kinoshita’s (2008) claim that the same-different matching task provides a pure measure of prelexical effects in masked priming.

### Method

The study (hypothesis, analyses, exclusion criteria, and sample size justification) was preregistered at OSF

#### Participants

Participants were the same as in Experiment 1.

#### Materials

The pool of word targets was the same as in Experiment 1 – they acted as “same” probe-target trials. Participants were exposed to 120 targets (60 high-frequency and 60 low-frequency targets) not seen in the lexical decision task (Experiment 1). In addition, we selected 240 additional words to act as “different” probe-target trials. The average Zipf frequency of these words was 4.16 (range 3.74–4.61), and the average OLD20 was 1.41 (range 1–1.95) in the subtitle corpus of the EsPal Spanish database (Duchon et al., 2013).

#### Procedure

In each trial of the same-different matching task, a reference string (i.e., probe) printed in lowercase was presented above a forward mask created by overlapping CAPTCHA-like items (as in Experiment 1) for 300 ms. Next, the probe disappeared, and the forward mask was replaced by a lowercase printed/CAPTCHA-like prime for 50 ms, which in turn was replaced by a target presented in uppercase. The target stimulus remained on the screen until the participant's response or 2 seconds had passed (in case of not responding before the timeout, the response was categorized as an error) (see Fig. 1b). Participants were instructed to decide whether the pairs of words were the same or not by pressing the “M” key (same) or the “Z” key (different) on the computer keyboard with their index fingers. They were told to make this decision as fast and as accurately as possible. Before each experimental block, there were 16 practice trials with the same manipulation as in the experimental trials. Participants were assigned to the appropriate counterbalanced list, ensuring that the items seen in Experiment 1 were not seen in Experiment 2 by the same participant. The sequence of the trials was randomized. This task took approximately 13–15 min.

### Results and discussion

Error rates were 5.64% for the “same” trials and 3.47% for “different” trials. As usual with this task, the focus was only on “same” trials since that is where the priming effect can be observed. RTs below 250 ms (0.14%) were excluded from analyses of the correct RTs. Table 3 shows the mean RT and error rates in each condition. Tables 4 (below) and 6 (OSM) present the estimates of the posterior distributions for the RT and accuracy analyses, respectively.

**Table 3:** Mean correct response times (RTs, in ms), percent error rates, and standard errors (SEs, in brackets) for words in Experiment 2 (masked priming same-different matching task)

		High-frequency		Low-frequency	
		RT (SE)	% Error (SE)	RT (SE)	% Error (SE)
Printed	Related	454 (5.91)	2.4% (0.966)	463 (6.21)	2.5% (0.963)
	Unrelated	530 (6.61)	11.1% (1.91)	537 (6.31)	11.8% (1.95)
Captcha	Related	465 (5.86)	2.5% (0.966)	474 (6.25)	2.8% (1.02)
	Unrelated	501 (6.29)	5.9% (1.46)	508 (6.01)	6.0% (1.47)
Printed	Priming effect	76	8.7%	74	9.3%
Captcha		36	3.4%	34	3.2%

*Note:* Standard errors were within-participant SEs around the mean (Cousineau & O’Brien, 2014)

**Table 4:** Posterior estimates parameters, estimation errors, and 95% credible intervals for the fixed effects of the model fitted for the response times to word targets in the same-different task (“same” trials, Experiment 2)

Parameters	Estimation	Estim. Error	Lower bound	Upper bound
Intercept (μ)	490.34	3.51	483.48	497.34
Intercept (β)	4.41	0.02	4.37	4.46
**Relatedness (**μ**)**	**55.08**	**1.78**	**51.55**	**58.58**
**Format (**μ**)**	**8.82**	**1.19**	**6.51**	**11.18**
**Word-Frequency (**μ**)**	**7.60**	**1.45**	**4.77**	**10.48**
**Relatedness × Format (**μ**)**	**39.07**	**2.62**	**33.93**	**44.24**
Relatedness × Word-Frequency (μ)	-1.02	2.75	-6.32	4.36
Format x Word-Frequency (μ)	0.60	2.34	-4.00	5.15
Relatedness × Format × Word-Frequency (μ)	1.41	4.58	-7.60	10.49
**Relatedness (β)**	**0.11**	**0.02**	**0.08**	**0.14**
**Format (β)**	**0.04**	**0.02**	**0.01**	**0.07**
**Word-Frequency (β)**	**0.04**	**0.02**	**0.01**	**0.08**
**Relatedness × Format (β)**	**0.07**	**0.03**	**0.00**	**0.13**
Relatedness × Word-Frequency (β)	-0.05	0.03	-0.11	0.01
Format × Word-Frequency (β)	0.01	0.03	-0.05	0.07
Relatedness × Format × Word-Frequency (β)	0.00	0.06	-0.12	0.12

*Note.* The estimations in bold indicate that the 95% credible interval did not overlap with zero

#### Response times

As shown in Table 4, for the Gaussian component, the analysis revealed that the responses were faster for identity than for unrelated pairs, to high-frequency words than to low-frequency words, and to targets preceded by CAPTCHA-like primes than to printed primes. In addition, the identity-priming effect was greater for printed primes than CAPTCHA-like primes (76 vs. 36 ms, respectively; interaction, *b* = 39.07, 95% CrI [33.93, 44.24]). Critically, unlike the lexical decision experiment, there was no evidence of other interactions (all |*b*s| < 1.41). The OSM presents the distributional differences across conditions as delta plots.

For the exponential component, we found a similar pattern. We obtained evidence of effects of prime-target relation (*b* = 0.11, 95% CrI [0.08, 0.14]), prime format (*b* = 0.04, 95% CrI [0.01, 0.07]), and word frequency (*b* = 0.04, 95% CrI [0.01, 0.08]), together with an interaction between prime-target relation and format (*b* = 0.07, 95% CrI [0.00, 0.13]).

This experiment showed greater identity priming for printed than CAPTCHA-like primes. More importantly, unlike Experiment 1 (lexical decision), the same-different matching task did not reveal any indication of modulations stemming from word frequency, suggesting that the obtained identity-priming effects were primarily driven by bottom-up activation rather than lexical feedback. Indeed, the lexical component (using word frequency as a marker) was minimal, only 7 ms.

## General discussion

The identification of visually presented words is tolerant to some distortions in the input format. Two potential explanations can capture this phenomenon: bottom-up activation and top-down lexical feedback. To shed light on this theoretical issue, “one of the oldest debates in visual word recognition” (Carreiras et al., 2014, p. 90), the present experiments examined whether the initial stages of the lexical processing of briefly presented distorted words rely on bottom-up activation (i.e., whether letter detectors tolerate some perceptual variability in the input; LCD model: Dehaene et al., 2005) or whether letter encoding can also benefit from top-down lexical feedback (i.e., whether lexical feedback helps to normalize the visual input; interactive word recognition models: McClelland & Rumelhart, 1981, and successors, like the Multiple Read-Out Model (Grainger & Jacobs, 1996), the Dual Route Cascaded Model (Coltheart et al., 2001), or the Spatial Coding Model (Davis, 2010)). We designed two masked priming experiments, one using a task that appears to be sensitive to lexical feedback (lexical decision task) and the other using a task that was proposed to only engage prelexical processing (same-different matching task). We compared the identity-priming effect (unrelated vs. identity primes) for distorted (CAPTCHA-like) primes versus unaltered (printed) primes in high- versus low-frequency target words. A purely bottom-up approach would predict that the identity-priming effect for CAPTCHA-like primes would be independent of word frequency in the two experiments. In contrast, an interactive approach would predict that, for high-frequency target words, CAPTCHA-like primes benefit more from top-down feedback than low-frequency words in the task involving lexical processing (lexical decision experiment).

The lexical decision experiment (Experiment 1) showed that identity priming was greater for printed than for CAPTCHA-like primes, replicating the findings reported by Hannagan et al. (2012). More importantly, the difference in identity priming for printed primes compared to CAPTCHA-like primes was much smaller for high-frequency than low-frequency words (9 vs. 27 ms, respectively). Thus, the activation from high-frequency word units helped to overcome the cost caused by distorted – CAPTCHA-like – primes. This favors the idea that top-down lexical feedback can be observed even in the first stages of visual word recognition. Critically, the above interaction vanished with the same-different matching task (Experiment 2), in which we found greater identity-priming effects for printed than CAPTCHA-like primes independent of word frequency. Indeed, the overall word frequency effect was minimal in the same-different matching task (7 ms), reinforcing the claims that it primarily targets prelexical effects. Therefore, the present experiments illustrate how task instructions modulate the amount of top-down lexical feedback: in Experiment 1, the decision regarding whether the target stimulus is a word was based on lexicality, whereas in Experiment 2, the decision regarding whether the target stimulus matches the probe was based on letter identity.

Thus, when the task requires lexical access, top-down processes from the lexical level modulate lower levels of processing even in the initial stages of visual word identification. As a result, the reading cost caused by distorted stimuli plays a less prominent role for high- than for low-frequency words. This pattern challenges the models that assume mainly bottom-up processing (e.g., Dehaene et al., 2005). According to these models, visual orthographic information is processed through a series of hierarchically organized stages. Each stage would occur in a strictly feedforward manner and, in its most rigid form, sequentially. Thus, the processing of distorted primes would not vary depending on higher-level features, like word frequency. Note that, being primarily bottom-up, the LCD model (Dehaene et al., 2005) also assumes that there may be a role for some feedback from higher processing layers in visual word recognition (see Dehaene & Cohen, 2007). As Qiao et al. (2010) indicated, this could be the case via extra attentional engagement when participants *consciously* identify heavily distorted words (e.g., poorly written handwritten words; see also Vergara-Martínez et al., 2021). However, it is unclear how this attentional mechanism could operate in a masked priming task when all target words are presented in pristine printed format.

Conversely, the present results can be easily accommodated in the framework of interactive models (McClelland & Rumelhart, 1981; see also Coltheart et al., 2001; Davis, 2010; Grainger & Jacobs, 1996): information would flow continuously (and bidirectionally) through the entire orthographic–phonological–lexical–semantic network. This process allows partially formed higher-level representations to provide feedback and influence lower-level representations in the network, such as perceptual features or orthography. In lexically-based tasks, the distorted information from the perceptual level flows bottom-up to the lexical representation of words, while lexical information (word frequency) from higher levels simultaneously flows top-down, facilitating the encoding. In other words, the (high-level) lexical information from high-frequency words helps the encoding of distorted (low-level) visual features.

Notably, the presence of top-down lexical feedback in word recognition is likely not confined to the visual modality; similar processes may also operate in the auditory domain. For instance, Dufour and Grainger (2020) found that word frequency modulates the transposed-phoneme effect in spoken-word recognition, which they interpreted as supporting top-down lexical feedback within an interactive activation framework (see Magnuson et al., 2024, for modeling lexical feedback in the TISK model of spoken-word recognition).

Finally, the present experiments also have methodological implications. The contrasting pattern of results observed between the lexical decision task and the same-different matching task highlights the importance of selecting the appropriate task based on the research question being addressed, as different methodologies can shed light on different aspects of word recognition. The (masked priming) lexical decision task is particularly useful for examining higher-level word processes (i.e., lexical access) and their interaction with lower – perceptual, orthographic – processes. In contrast, the (masked priming) same-different matching task is better suited for isolating and investigating lower-level word processing (i.e., perceptual and orthographic factors). Thus, as experimenters, we must make assumptions about how word recognition manifests in the experimental task. Paraphrasing Nietzsche, our research tools are also working on our processes (real quote in Kittler, 1999).

In sum, we demonstrated that even in the initial phases of lexical processing during word recognition, higher-level linguistic processes influence lower-level perceptual processes. Further experimentation using measures with better temporal resolution (e.g., evoked response potentials (ERPs)) may provide direct insight into the internal temporal dynamics underlying how different representations are activated.

## Supplementary Information

Below is the link to the electronic supplementary material.

Supplementary file1 (PDF 202 KB)

## Data Availability

The datasets generated and/or analyzed during the current study are available in OSF: https://osf.io/9qbj2/?view_only=221015adf0c24525a33abe5588384a37.

## Footnotes

1 While not the focus of the present experiment, it is often the case that, behaviorally, masked identity priming is greater for low- than high-frequency words (e.g., Kinoshita, 2006). This pattern can be explained because the activation levels of low-frequency words, being slower than high-frequency, leave more room for the repetition priming by the time of reaching the criterion for "word" responses (see Grainger et al., 2012).

2 The Zipf frequency is a logarithmic measure of word frequency in a corpus, where the value indicates how common or rare a word is, based on Zipf's Law. It compresses word frequency values into a scale from 1 (rare words) to 7 (extremely common words) (see van Heuven et al., 2014). The OLD20 (Orthographic Levenshtein Distance) is a psycholinguistic measure of word similarity. It represents the average number of single-letter changes (insertions, deletions, or substitutions) needed to transform a given word into its 20 nearest orthographic neighbors (see Yarkoni et al., 2008).

3 Using both visual and auditory presentations in the same-different matching task, Kelly et al. (2013) found comparable masked orthographic priming effects for words and nonwords in the visual modality, whereas orthographic priming occurred only for words when the reference was presented auditorily. They interpreted this pattern as reflecting that masked priming effects in same-different matching tasks may be sensitive to lexical influences under certain conditions (i.e., auditorily presented stimuli).
