# Supplementary material for: Top-down feedback normalizes distortion in early visual word recognition: Insights from masked priming
Source: Psychon Bull Rev. 2024 Oct 18;32(2):920–9. doi: 10.3758/s13423-024-02585-2 (PMC12000148; doi:10.3758/s13423-024-02585-2)
Supplement: Supplementary file 1 — Supplementary file1 (PDF 202 KB) [file 13423_2024_2585_MOESM1_ESM.pdf]

Supplemental materials

Experiment 1: Masked priming lexical decision task

Error rates were 5.58% for word trials and 4.66% for nonword trials. We focused on the word targets because our manipulation relied on whether the identity priming effects for the captcha and printed primes interacted with a lexical factor (word frequency). Response times below 250 ms (155 data points, 0.27%) were excluded from analyses of the correct response times. Table 1 (of the ms) displays the mean response time and error rates in each condition, and Table 2 (of the ms) presents the estimates of the posterior distributions for the reaction times. The accuracy analyses are available in Table 5.

Table 5. Posterior estimates parameters, Estimation errors, and 95% Credible Intervals for the fixed effects of the model fitted for the accuracy to word targets in the lexical decision task (Experiment 1).

Parameters	Estimation	Estim. Error	Lower bound	Upper bound
Intercept	3.73	0.09	3.55	3.92
**Relatedness**	**-0.63**	**0.10**	**-0.83**	**-0.43**
Format	-0.08	0.08	-0.08	0.25
**Word-Frequency**	**-1.71**	**0.16**	**-2.03**	**-1.39**
Relatedness x Format	-0.31	0.17	-0.65	0.03
Relatedness x Word-Frequency	-0.06	0.18	-0.41	0.28
Format x Word-Frequency	-0.03	0.15	-0.33	0.27
Relatedness x Format x Word-Frequency	0.14	0.31	-0.45	0.76

Note. The estimations in bold indicate that the 95% Credible Interval did not overlap with zero.

The accuracy analysis showed that responses to identity prime-target pairs were 3.55% more accurate than responses to unrelated pairs (relatedness effect; b = -0.63, 95% CrI [-0.83, -0.43]). In addition, responses to high-frequency words were 5.7% more accurate than responses to low-frequency words (frequency effect; b = -1.71, 95% CrI [-2.03, -1.39]). There was no evidence of other main effects or interactions (all $|bs| < 0.31$).

Experiment 2: Masked priming same different matching task

Error rates were 5.64% for the "same" trials and 3.47% for "different" trials. As usual with this task, the focus was only on "same" trials since that is where the priming effect can be observed. Response times below 250 ms (81 data points, 0.14%) were excluded from analyses of the correct RTs. Table 3 of the ms shows the mean response time and error rates in each condition. Tables 4 (of the ms) and 6 (below) present the estimates of the posterior distributions for the RT and accuracy analyses, respectively.

Table 6. Posterior estimates parameters, Estimation errors, and 95% Credible Intervals for the fixed effects of the model fitted for the accuracy to word targets in the same-different task ("same" trials, Experiment 2).

Parameters	Estimation	Estim. Error	Lower bound	Upper bound
Intercept	3.30	0.06	3.18	3.43
**Relatedness**	**-1.27**	**0.09**	**-1.45**	**-1.10**
**Format**	**-0.34**	**0.07**	**-0.48**	**-0.20**
Word-Frequency	-0.05	0.08	-0.20	0.10
**Relatedness x Format**	**-0.67**	**0.16**	**-0.97**	**-0.35**
Relatedness x Word-Frequency	0.02	0.14	-0.26	0.30
Format x Word-Frequency	0.02	0.13	-0.24	0.28
Relatedness x Format x Word-Frequency	-0.20	0.28	-0.76	0.35

Note. The estimations in bold indicate that the 95% Credible Interval did not overlap with zero.

The accuracy analysis showed an interaction between prime-target relatedness and prime format (b = -0.67, 95% CrI [-0.97, -0.35]): responses to captcha-like primes were more accurate than responses to printed primes (the error rates were 5.9% and 11.1%, respectively) but this only occurred for unrelated primes (there were virtually no differences with identity prime-target pairs (error rates were 2.5% and 2.4% for captcha-like and printed primes, respectively).
